# Supplementary material for: Inflammatory factors and risk of lung adenocarcinoma: a Mendelian randomization study mediated by blood metabolites
Source: Front Endocrinol (Lausanne). 2024 Aug 27;15:1446863. doi: 10.3389/fendo.2024.1446863 (PMC11384989; doi:10.3389/fendo.2024.1446863)
Supplement: Supplementary file 1 [file DataSheet1.zip › supplementary files/S2/GCST90200800/forest.pdf]

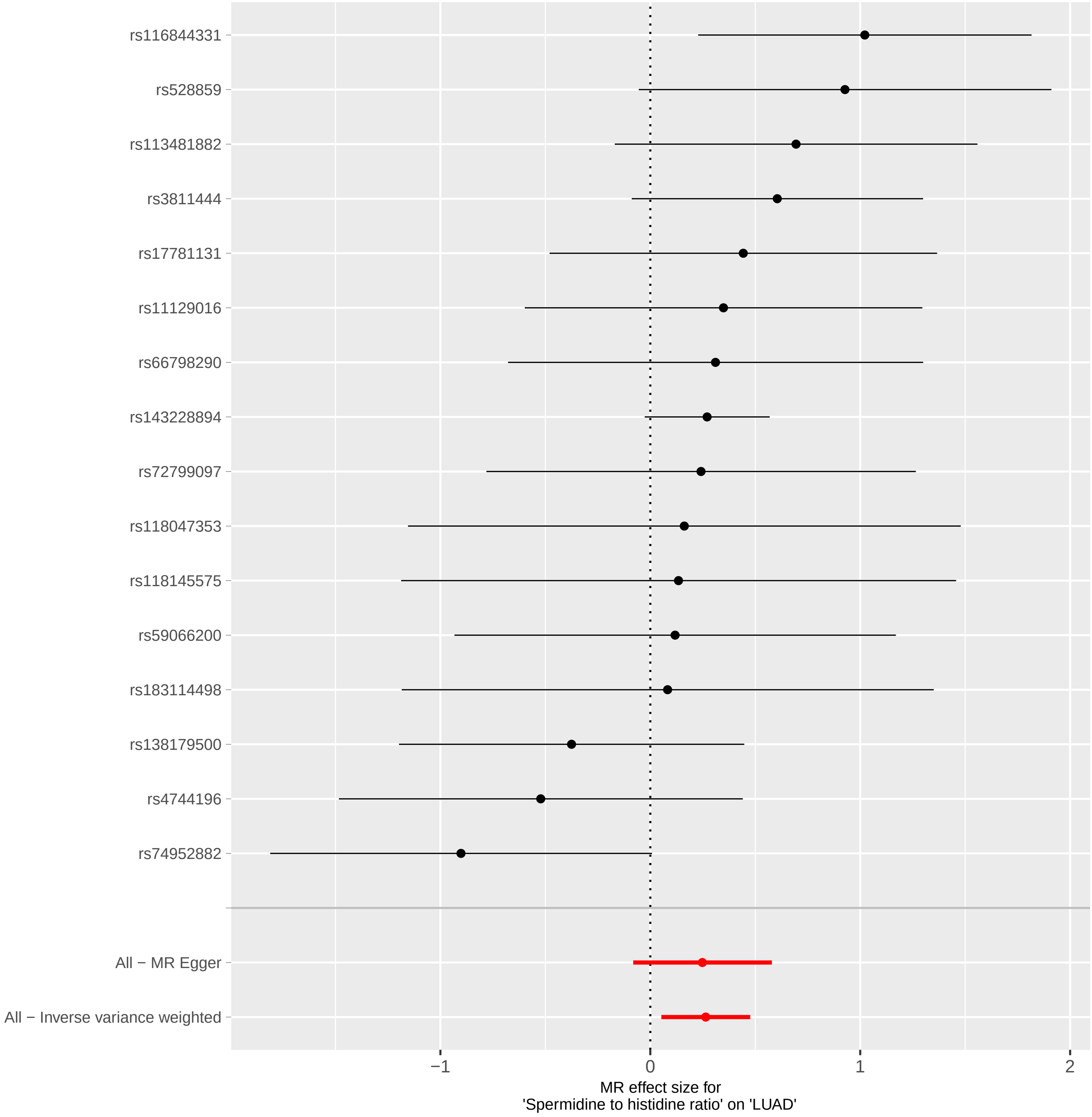

rs116844331
rs528859
rs113481882
rs3811444
rs17781131
rs11129016
rs66798290
rs143228894
rs72799097
rs118047353
rs118145575
rs59066200
rs183114498
rs138179500
rs4744196
rs74952882
All − MR Egger
All − Inverse variance weighted
−1
0
1
2
MR effect size for
'Spermidine to histidine ratio' on 'LUAD'
